# Nitric Oxide Donor Spermine-NONOate Elicits Endogenous Dispersal-Associated Transcriptional Responses to Promote Biofilm Dispersal in *Pseudomonas aeruginosa*

**DOI:** 10.3390/antibiotics15030278

**Published:** 2026-03-09

**Authors:** Xavier Bertran Forga, Kathryn E. Fairfull-Smith, Jilong Qin, Makrina Totsika

**Affiliations:** 1 Centre for Immunity and Infection Control, School of Biomedical Sciences, Queensland University of Technology, Brisbane, QLD 4000, Australia; 2 Max Planck Queensland Centre, Queensland University of Technology, Brisbane, QLD 4000, Australia; 3 School of Chemistry and Physics, Queensland University of Technology, Brisbane, QLD 4000, Australia; 4 Centre for Materials Science, Queensland University of Technology, Brisbane, QLD 4000, Australia; 5 Institute for Molecular Bioscience, The University of Queensland, Brisbane, QLD 4072, Australia

**Keywords:** *Pseudomonas aeruginosa*, biofilm dispersal, nitric oxide, nitroxides, C-TEMPO, RNA-seq, transcriptomics, ANR, dispersal biomarkers

## Abstract

**Background/Objectives**: Bacterial biofilms are structured communities of sessile cells embedded in a self-produced extracellular matrix. Within biofilms, bacteria become highly tolerant toenvironmental stressors such as host immune responses and antimicrobial treatments. In response to specific cues, however, biofilm cells can revert to a planktonic free-swimming lifestyle through a process termed biofilm dispersal. When dispersed cells escape the biofilm matrix, they lose biofilm-associated antibiotic tolerance, a major barrier to treating medical biofilms. As such, dispersal-inducing compounds like nitric oxide (NO) are actively investigated as adjuvants to potentiate the biofilm-eradicating activity of existing antibiotics. We recently characterised the transcriptomic responses elicited during spontaneous biofilm dispersal in closed culture-grown *Pseudomonas aeruginosa* biofilms. Here, we evaluated the transcriptional profiles of *P. aeruginosa* biofilms treated with the NO donor Spermine-NONOate (SP-NONO) and the nitroxide C-TEMPO, an NO analogue, to determine potential pathways involved in NO-mediated dispersal. **Methods**: Dispersal activity on *P. aeruginosa* PAO1 biofilms by SP-NONOate and C-TEMPO was quantified by crystal violet staining. Cellular responses to each compound were profiled by RNA-seq on treated and untreated cells. **Results**: While both compounds disrupted the transcription of ANR-regulated energy metabolism pathways, only SP-NONO activated canonical NO-regulated responses. Considering that only SP-NONO showed biofilm dispersal activity in this culture system, we investigated shared transcriptional shifts in SP-NONO-treated and spontaneously dispersed biofilms to identify pathways likely involved in central dispersal responses. These mostly included genes involved in the catabolism of branched-chain amino acids (leucine, valine, isoleucine) and lysine, as well as 9 of 14 genes previously defined as transcriptional biomarkers of spontaneous biofilm dispersal. **Conclusions**: This study suggests that NO disrupts biofilm maturation by prematurely stimulating central pathways of spontaneous biofilm dispersal and highlights this set of biomarkers as robust indicators of dispersal responses.

## 1. Introduction

Bacterial biofilms are aggregates of sessile cells, typically attached to surfaces, and encased within an extracellular matrix [1]. During biofilm maturation, cells acquire an elevated tolerance to environmental stress, host immune defences and antibiotics [2]. As part of their life cycle, mature biofilms naturally undergo biofilm dispersal, activated by environmental or self-synthesised cues [3,4]. During dispersal, biofilm-residing cells regain planktonic attributes to resume a free-swimming lifestyle [5], while simultaneously losing their biofilm-associated antibiotic tolerance [6]. This makes dispersal-inducing compounds a promising strategy to combat chronic biofilm infections through the potentiation of antibiotics that are otherwise ineffective against biofilms.

**Figure 1:**
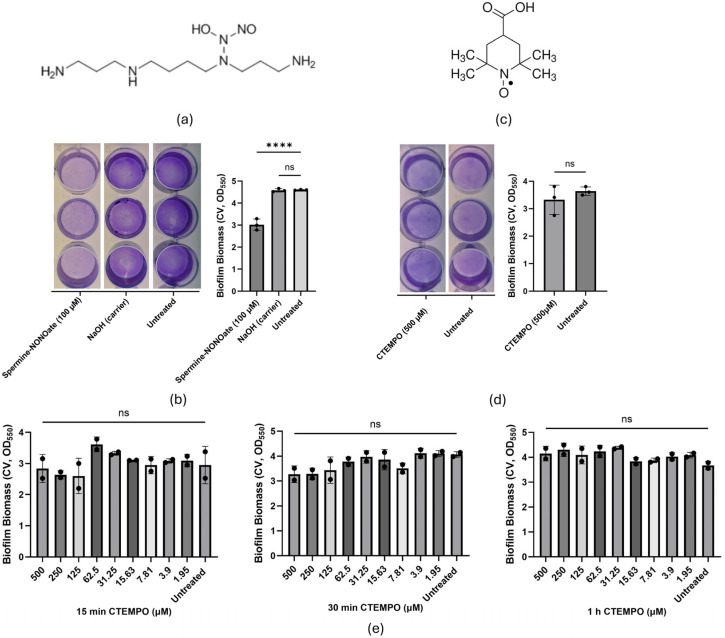
Biofilm responses of *P. aeruginosa* PAO1 to SP-NONO and C-TEMPO. Structures of (**a**) SP-NONO and (**c**) C-TEMPO are depicted, including the delocalised electron (•) in the nitroxide moiety of C-TEMPO. *P. aeruginosa* PAO1 cultures were seeded on 24-well plates, and biofilm biomass was quantified by crystal violet (CV) staining (OD_550nm_) after no treatment or treatment with (**b**) SP-NONO (100 µM, 15 min), and NaOH (vehicle control; 100 µM) or (**d**) C-TEMPO (500 µM, 30 min). Images of stained biofilms and dot plots of CV quantification are shown for 3 biological replicates, with means ± SD also shown in the graphs. Statistical differences between groups were calculated by Ordinary One-Way ANOVA with Dunnett’s test for SP-NONO treatments relative to Untreated control and by Unpaired two-tailed Student’s *t*-test for C-TEMPO treatments (****, *p* value < 0.0001). (**e**) Treatment with C-TEMPO (500–1.95 µM) over 15, 30 or 60 min; 2 biological replicates were included. Means ± SD are represented in the graphs. Statistical differences between groups were calculated by Ordinary One-way ANOVA with Dunnett’s test (ns = not significant).

The water-soluble radical gas nitric oxide (NO) has gained attention as one of the most promising dispersal agents, showing broad-spectrum effects on Gram-positive and Gram-negative bacteria [7,8]. In the opportunistic biofilm-forming bacterium *Pseudomonas aeruginosa,* the exogenous addition of NO-releasing compounds (NO donors) such as Spermine-NONOate (SP-NONO; Figure 1a) induced rapid reductions (~40–88%) in the biomass of surface-attached biofilms [9,10,11,12,13]. Furthermore, NO gas and NO donors have proven effective in the dispersal of highly recalcitrant *P. aeruginosa* isolates from cystic fibrosis patients in vitro, and reduced the bacterial load in their sputum when used adjunctively with antibiotics [8,12]. In *P. aeruginosa*, these responses are associated with the activation of pathways leading to the reduction of c-di-GMP, the intracellular second messenger controlling bacterial lifestyle, leading to increased motility through rhamnolipid production and reduced transcription of matrix exopolysaccharides [14,15,16,17]. Notably, dispersal occurs despite *P. aeruginosa* possessing distinct NO-responsive transcriptional regulators that tightly control the expression of detoxification mechanisms to prevent NO-derived toxicity. These NO-detoxification regulators are the dissimilatory nitrate respiration regulator DNR, and FhpR, a homolog of the *E. coli* transcriptional regulator NorR [10,18,19], which respectively regulate the expression of the NO reductase NorBC and the flavohaemoglobin Fhp [20,21].

Despite preclinical efficacy, the clinical application of NO remains challenging, as its small size, high diffusion capacity and pronounced chemical reactivity result in a short biological half-life and complicate controlled, localised delivery at the site of infection [22]. Nitroxides are considered NO analogues due to sharing key chemical features with NO while presenting significantly reduced reactivity [23]. Like NO, nitroxides possess a delocalised unpaired electron over the nitrogen and oxygen atoms. However, this group is sterically hindered by adjacent methyl groups that limit their reactivity (e.g., 4-carboxy-TEMPO; Figure 1b) [23]. Nitroxides were shown in some studies to induce slow dispersal of *P. aeruginosa* biofilms grown under continuous flow conditions (open culture systems) and potentiate the bactericidal activity of antibiotics, thus highlighting their utility as antibiofilm agents [24,25].

Recently, we reported the biofilm culture kinetics of *P. aeruginosa* PAO1 in closed culture systems, showing they recapitulated the main stages of the biofilm life cycle (attachment, maturation and spontaneous dispersal) described under continuous flow culture conditions [26]. Moreover, by characterising the transcriptomic profile of cells undergoing each stage, we temporally resolved canonical stage-specific transcriptional responses, such as the activation of surface-sensing pathways during attachment, polysaccharide production during biofilm maturation and quorum sensing signals during dispersal [26]. Notably, the upregulation of fourteen genes was associated with the onset of rapid loss of biofilm biomass, and this set was thus proposed to serve as specific biomarkers of biofilm dispersal [26]. Dispersal biomarkers included genes encoding transcriptional regulators *amrZ* (PA3385) and *cdpR* (PA2588); chemotaxis- and redox-associated genes *cheR2* (PA0175), *pqqA* (PA1985) and PA0743; fimbrial- and cupin-mediated adhesion loci *tadA* (PA4302), *rcpA* (PA4304), *rcpC* (PA4305), *flp* (PA4306) and *cupE1/E2* (PA4648 and PA4649); and uncharacterised genes PA0111, PA1353 and PA4523. Whether biofilm dispersal agents, like NO and nitroxides, activate transcriptional responses that govern spontaneous biofilm dispersal has not been investigated to date.

Here, we assessed the dispersal activity of NO donor Spermine-NONOate (SP-NONO) and nitroxide 4-carboxy-TEMPO (C-TEMPO) using microplate-grown *P. aeruginosa* biofilms. Subsequently, we performed RNA sequencing on biofilm cells treated with each compound and characterised their transcriptional profiles relative to spontaneous dispersal. Specifically, we report that >50% of genes differentially regulated under SP-NONO treatment were found similarly altered during spontaneous dispersal, and that SP-NONO, but not C-TEMPO, upregulated most transcriptional biomarkers associated with spontaneous dispersal in 12-hour-old biofilms. These findings suggest that NO prematurely activates spontaneous dispersal pathways to induce dispersal of *P. aeruginosa* biofilms. Collectively, our data indicate that these biomarkers robustly represent central pathways involved in the activation of dispersal responses in *P. aeruginosa*.

## 2. Results

### 2.1. The NO Donor SP-NONO, but Not the Nitroxide C-TEMPO, Rapidly Induced Biofilm Biomass Reduction of P. aeruginosa Biofilms in Closed Systems

To determine the treatment durations of SP-NONO and C-TEMPO required to induce biofilm dispersal, and to define appropriate time points for harvesting mRNA to assess gene expression changes, we performed biofilm dispersal assays using both compounds. In closed culture systems such as microtiter plates, *P. aeruginosa* PAO1 rapidly forms biofilms within 4 h of inoculation [13,26]. Accordingly, the dispersing activities of the C-TEMPO and SP-NONO were assessed at sub-inhibitory concentrations on *P. aeruginosa* biofilms grown for 4 h (Figure S1A). In this experimental model, dispersal is reported as a reduction in attached biofilm biomass, quantified by crystal violet staining at 550 nm of optical density, which we demonstrated to correlate with decreased biofilm-embedded cells after treatment with NO donors [13]. Consistent with our previous work [13], both untreated biofilms and mock-treated biofilms (NaOH vehicle control) showed high biomass (OD_550_~4.5), whereas biofilms treated with SP-NONO (100 µM, 15 min) exhibited significantly reduced biomass (OD_550_~3, Figure 1a). Based on these results, biofilm cells treated with SP-NONO (100 µM) for 15 min were used for downstream RNA-seq to characterise the transcriptional responses induced by this biofilm-dispersing NO donor.

To assess the dispersal activity of C-TEMPO in closed culture-grown *P. aeruginosa* PAO1 biofilms, we initially treated biofilms for 15 min (to match SP-NONO treatment) with a broad range of concentrations (500–1.95 µM; Figure 1e). No biomass changes were observed after 15 min of C-TEMPO treatment, or when treatment time was extended to 30 min or 1 h (OD_550_~3.5; Figure 1b,e). In contrast to previous reports using flow cell systems, these results indicate that C-TEMPO does not elicit detectable dispersal activity in closed biofilm cultures [24,27]. We reasoned that the divergent effects on biofilms displayed by two representatives of analogue compound families (NO donors and nitroxides) could be leveraged to identify common transcriptional variations induced by SP-NONO and nitroxides and, therefore, discriminate unique transcriptional responses by SP-NONO to refine candidate pathways associated with dispersal. Consequently, RNA-seq samples were collected from biofilm *P. aeruginosa* PAO1 cells treated with C-TEMPO (500 µM) for 30 min. This extended exposure reflects the distinct physicochemical properties of the compounds, accounting for the bulkier structure of C-TEMPO relative to NO (Figure 1a,c), which likely reduces the penetration kinetics of the compound. On the other hand, treatment durations were limited to 30 min, due to *P. aeruginosa* PAO1 biofilm culture kinetics rapidly progressing through stages in closed systems [26], suggesting that a longer treatment would risk confounding transcriptional differences arising from biofilm ageing.

### 2.2. SP-NONO and C-TEMPO Disrupt ANR-Regulated Energy Production Pathways

**Figure 2:**
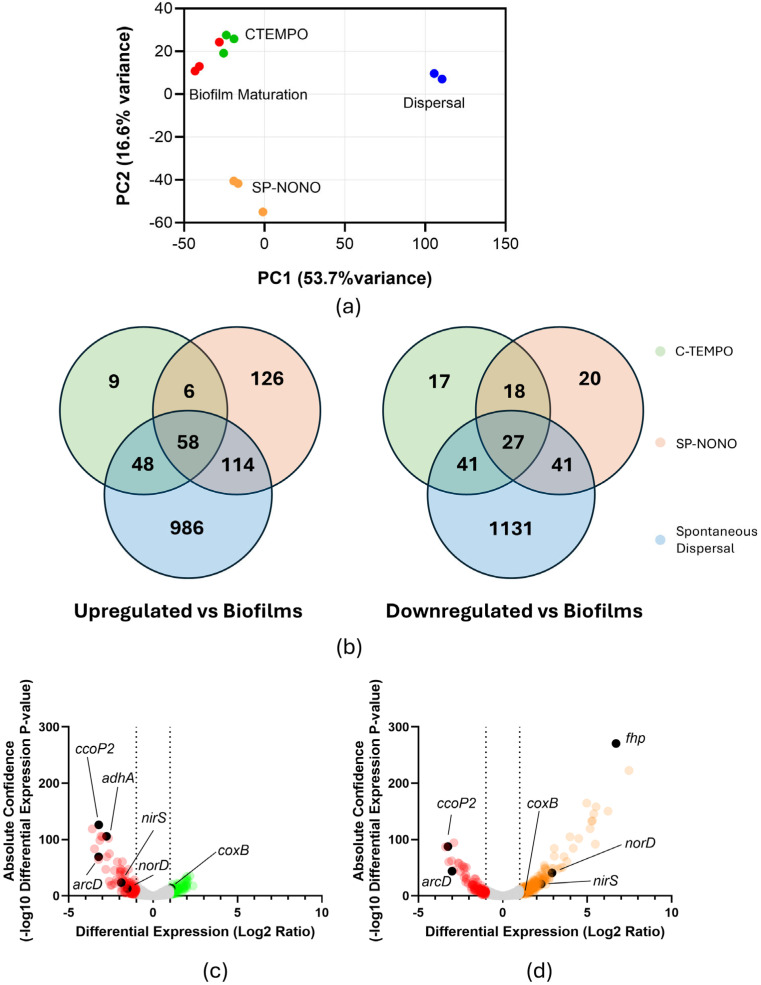
Transcriptional profiles of C-TEMPO-treated and SP-NONO-treated *P. aeruginosa* biofilms. (**a**) Principal Component Analysis of RNA-Seq data from biofilm-residing cells treated with SP-NONO (orange) or C-TEMPO (green). Data were plotted with RNA-seq data from previously obtained untreated mature biofilm-residing cells (red) or spontaneously dispersed cells (blue) [26]. Normalised read counts for each gene (RPKM) were extracted from each sample and transformed into PC loadings using ClustVis. The values of PC1 and PC2 of each sample, accounting for the largest variance, are represented. Each point in the graph represents a biological replicate (SP-NONO, n = 3; C-TEMPO, n = 3; biofilm-residing cells, n = 3; spontaneously dispersed cells, n = 2). (**b**) Venn diagrams of differentially transcribed genes using the comparisons C-TEMPO (green), SP-NONO (orange) and Spontaneous dispersal (blue) relative to untreated biofilms. (**c**,**d**) Volcano plot of genes upregulated and downregulated after C-TEMPO (green) and SP-NONO (orange) treatments relative to untreated biofilms (red). A full list of genes and their respective expressions relative to untreated 4-hour-old biofilms can be found in Table S1.

To compare the global transcriptional profiles between treated and untreated groups, RNA-seq data was plotted using ClustVis (Figure 2a). C-TEMPO-treated biofilms (Figure 2a—green) clustered closely with untreated biofilm-residing cells (Figure 2a—red) obtained from earlier work [26], indicating that C-TEMPO treatment induced minimal transcriptional changes in biofilms. In contrast, SP-NONO-treated biofilms (Figure 2a—orange) clustered separately from untreated biofilms, indicating that a significant transcriptional shift was induced. To quantify these responses, we used a negative binomial test (*p*-value ≤ 0.01; Log_2_ fold-change ≥ |1|) comparing NO-treated or C-TEMPO-treated samples to untreated, 4 h biofilm cells.

Treatment of biofilms with C-TEMPO altered the transcription of 224 genes (121 upregulated; 103 downregulated), with downregulated genes exhibiting markedly greater absolute fold changes compared to upregulated genes (Figure 2c), whereas SP-NONO treatment affected the transcription of 407 genes (304 upregulated; 103 downregulated) (Figure 2b,d). Despite that C-TEMPO treatment did not change biofilm biomass, unlike SP-NONO, we observed a substantial overlap in transcriptional responses under C-TEMPO and SP-NONO treatments, with 64/121 C-TEMPO-upregulated genes and 45/103 C-TEMPO-downregulated genes also similarly regulated by SP-NONO (Figure 2). The genes with the largest transcriptional differences shared between C-TEMPO and SP-NONO were involved in ANR-controlled oxidative phosphorylation and energy production (Table 1). In contrast, genes under regulation from dedicated NO sensors were uniquely upregulated by SP-NONO. These included genes encoding elements of the denitrification pathway (Table 1) or the aerobic NO detoxification flavohaemoprotein Fhp (104-fold, Table S1). Collectively, the observed differences in gene expression suggest that C-TEMPO overlaps with SP-NONO in disrupting O_2_-mediated transcriptional regulation, while being unable to induce canonical responses to NO.

### 2.3. SP-NONO Upregulates Metabolic Pathways of Spontaneous Dispersal

In a previous study, we identified the transcriptional profiles of *P. aeruginosa* in each stage of the biofilm life cycle, including attachment, biofilm maturation and, more importantly, spontaneous dispersal (Figure 2a—green) [26]. Considering that SP-NONO elicited significant reduction in biofilm biomass analogous to that observed during spontaneous dispersal (Figure 1c), we hypothesised that central responses involved in dispersal may be reflected in the transcriptional profiles of cells treated with SP-NONO. Indeed, we found 176/304 genes upregulated and 68/103 genes downregulated by SP-NONO that overlapped with transcriptional changes occurring during spontaneous dispersal (Figure 2b). To identify gene expression changes strictly associated with the dispersal phenotype, genes uniquely upregulated by SP-NONO and during spontaneous dispersal, but not by C-TEMPO, were compiled into Table 2 (a full list of genes was included in Table S2). Transcriptomic profiles associated with biofilm dispersal displayed a downregulation of genes predicted to be involved in the import of sulphur-containing metabolites such as sulphate (*cysW*, *cysT* and *cysP*) and taurine (PA3936–PA3938), based on KEGG pathway annotation [27]. *cobP*, *cobU* and *cobV*, involved in the cobalamin biosynthesis pathway, were similarly downregulated (Table 2) [28]. In contrast, pathways associated with energy generation were upregulated. These included genes involved in the catabolic degradation of amino acids such as lysine to glutarate (*davD* and *davT*) [29], or of valine, leucine and isoleucine into precursors of the TCA cycle (*braC*, *bkdB*, *lpdV*, PA3417 and *ldh*) (Table 2) [30,31], as well as genes involved in pyrroloquinoline quinone biosynthesis (*pqqA*, *pqqD*, *pqqE* and *pqqF*) (Table 2), a cofactor required for the periplasmic oxidation of ethanol [32]. Moreover, cells undergoing SP-NONO treatment or spontaneous dispersal promoted the upregulation of genes belonging to the Che2 chemotaxis system (PA0173–PA0179) (Table 2) [33], suggesting a role of this chemosensory pathway in biofilm dispersal.

### 2.4. SP-NONO Treatment of PAO1 Biofilms Upregulates Biomarkers of Spontaneous Dispersal

By analysing the temporal changes in global gene expression across the biofilm life cycle stages, we previously identified fourteen transcriptional biomarkers that are distinctly and reproducibly upregulated during spontaneous dispersal in 12-hour-old biofilms [26]. Importantly, the dispersal-inducing NO donor SP-NONO largely recapitulated this distinct transcriptional response of spontaneous dispersal, whereas C-TEMPO did not (Table 3). Of the fourteen dispersal biomarkers, SP-NONO significantly increased the transcription of nine. These included PA0111 (6.62-fold), *cheR2* (3.41-fold)*, pqqA* (2.52-fold), *tadA* (2.21-fold), *rcpA* (2.12-fold), *rcpC* (2.18-fold), *flp* (2.27-fold) and *cupE1E2* (2.36- and 2.09-fold, respectively) (Table 3). In contrast, C-TEMPO only upregulated three biomarkers: PA0111 (5.15-fold), *tadA* (2.01-fold), and *flp* (2.20-fold) (Table 3). The observed transcriptional overlap suggests that SP-NONO signals the upregulation of pathways activated during spontaneous dispersal to prematurely elicit a reversion to the planktonic lifestyle. Furthermore, the upregulation of this set of genes during exogenously induced and spontaneous dispersal (reported as reduced surface-attached biofilm biomass) in closed culture systems strongly supports them as biomarkers representative of central dispersal pathways in *P. aeruginosa*.

## 3. Discussion

Biofilm dispersal agents have gained increasing attention as a therapeutic approach to enhancing antibiotic activity against clinical biofilms, which are the source of ~80% of chronic hospital infections. Biofilm infections show elevated incidence in prostheses and in-dwelling devices, leading to bacteraemia, ventilator-associated respiratory infections and catheter-associated urinary tract infections [34,35]. Among biofilm-forming bacterial pathogens, *P. aeruginosa* stands out due to causing ~7% of all healthcare-associated infections, with incidence rates as high as ~23% in ICU infections and in cystic fibrosis patients, causing highly recalcitrant endobronchiolitis, bronchiectasis, and pneumonia [36,37]. To fast-track screening of dispersal-inducing compounds, we previously identified a subset of *P. aeruginosa* genes that serve as transcriptional biomarkers of the onset of spontaneous dispersal [26]. Here, we demonstrate the robustness of these biomarkers using the biofilm dispersal NO donor SP-NONO, which effectively dispersed *P. aeruginosa* biofilms and elicited the upregulation of 9 of the 14 biomarkers. In contrast, the nitroxide C-TEMPO, which did not promote biofilm dispersal, only stimulated the upregulation of three dispersal biomarkers.

As a nitroxide, C-TEMPO possesses a significantly bulkier structure than NO and is sterically hindered by the four adjacent methyl groups. This implies significantly reduced reactivity and penetration relative to NO [23], which we addressed by testing a range of concentrations and treatment times. Nevertheless, no significant changes in attached biomass were observed in microplate-grown biofilms by any C-TEMPO treatment tested. Our data contrast with previous reports of C-TEMPO-mediated biofilm dispersal of *P. aeruginosa* in flow cells [24,25]. Importantly, *P. aeruginosa* biofilms were cultured for 48 h prior to being treated with C-TEMPO for a further 24 h, when an increased cell density was detected in the culture effluent [25]. Under flow culture conditions, the constant stream of media replenishes nutrients while removing metabolic by-products and quorum sensing molecules [38]. Therefore, biofilm maturation may span days, leading to the formation of niches within the multicellular community [25,39]. This is supported by reports indicating that ratio of exopolysaccharides to biofilm cell density is enhanced in *Pseudomonas fluorescens* under flow conditions relative to biofilms in closed systems [39]. Additionally, shear forces under flow conditions promote *P. aeruginosa* surface attachment and c-di-GMP biosynthesis, leading to increased biofilm formation [40]. Longer maturation times under conditions promoting the synthesis of matrix components likely promotes physiological variations relative to younger biofilms under closed culture conditions, which would lead to the development of nitroxide-responsive subpopulations.

Contradictory dispersal activity across different biofilm models, however, is not unique to nitroxides. Previously, we reported that the NO donor sodium nitroprusside, which has been widely documented to disperse *P. aeruginosa* biofilms in continuous flow cultures, unexpectedly increased biomass of *P. aeruginosa* biofilms cultured in microtiter plates [13]. While no studies have directly compared transcriptomic or metabolomic differences in biofilms of the same strain cultured under different culture platforms, it is likely that longer incubation times in continuous flow cultures account for biological differences in *P. aeruginosa* biofilms, thus affecting the dispersal activity of some drug candidates.

Here, we report for the first time the transcriptional response of *P. aeruginosa* to C-TEMPO treatment. This analysis revealed pronounced changes in genes whose transcription is modulated by the transcriptional regulator of anaerobiosis ANR and the redox-responsive two-component regulator RoxSR, which were also identified under SP-NONO treatment. These include genes required for survival under conditions of hypoxia [41], such as the NO-sensitive regulator encoded by *dnr*, the operon *ccoP2-ccoO2* encoding the high-affinity cbb3-type cytochrome C oxidase, the aa3-type cytochrome encoded by *coxAB*, and *cioAB*, encoding a cyanide-insensitive terminal oxidase [42]. Additionally, the ANR-regulated operon, *arcDABC*, and *adhA* were among the most downregulated genes by C-TEMPO and SP-NONO. These genes respectively encode the arginine deiminase pathway for the degradation of arginine to ornithine and a NAD^+^-dependent alcohol dehydrogenase, which participate in processes necessary for ATP generation under anaerobiosis, and are defined as strongly regulated by ANR and DNR [41,43]. In contrast, our analysis revealed no observable overlap between the SP-NONO and C-TEMPO regarding NO-mediated transcriptional regulation. Neither genes involved in denitrification (including the NIR, NOR and NOS operons), nor the O_2_-dependent flavohaemoglobin Fhp were upregulated by C-TEMPO, whereas SP-NONO strongly stimulated their transcription. Hence, despite nitroxides being widely proposed to act as NO analogues, our data suggest that C-TEMPO would primarily interact with ANR-regulated pathways, and that these would not be directly involved in SP-NONO-mediated dispersal.

NO is a well-established biofilm dispersal agent, known to increase cell motility and stimulate the enzymatic hydrolysis of the cellular signalling molecule cyclic-di-GMP [14]. Here, we reported the overlapping transcriptomic responses of chemically and spontaneously dispersed *P. aeruginosa* PAO1 biofilms in closed cultures. In both datasets, genes involved in the catabolism of valine, leucine and isoleucine (*braC*, *bkdB*, *lpdV*, PA3417 and *ldh*) were upregulated, together with genes encoding enzymes mediating the degradation of the lysine metabolite δ-aminovalerate to glutarate (*davD* and *davT*) [44], and a δ-aminovalerate-ABC transporter (*agtABC* but not *agtD*) [45]. Additionally, spontaneously and SP-NONO-dispersed cells largely upregulated *dctA* encoding the C_4_-dicarboxylic acid transport for the primary import of succinate, malate and fumarate [46], and PA0752-PA0754 (*tctABC*), encoding a citrate and cis-aconitate import [47]. Metabolic pathways involved in amino acid catabolism have also been reported as upregulated in independent transcriptomic studies of dispersal using SP-NONO [9]. Namely, *bkdA1*, *bkdA2*, *bkdB* and *lpdV* were amongst the most upregulated genes in SP-NONO-dispersed cells relative to untreated biofilms [48], suggesting that the activation of amino acid catabolic pathways and transport systems for TCA cycle intermediates may be derived from a dispersal-induced metabolic shift aiding cell reversal to the planktonic lifestyle.

In closed systems, we report that treatment with the NO donor SP-NONO caused maturing *P. aeruginosa* biofilms to upregulate PA0111, *cheR2*, *pqqA*, *tadA*, *rcpA*, *rcpC*, *flp*, *cupE1* and *cupE2*. These were proposed as transcriptional biomarkers of dispersal, as their transcription was largely increased in biofilms undergoing spontaneous dispersal [26]. Here, we demonstrate that, despite 4-hour-old and 8-hour-old biofilms displaying markedly distinct transcriptomic profiles, treatment with SP-NONO prematurely induced the discrete upregulation of most transcriptional biomarkers of dispersal. Therefore, we here report an overlap between two transcriptional signatures corresponding to distinct modes of dispersal induction at different biofilm ages, indicating that NO promotes the upregulation of a core set of transcriptional biomarkers associated with the onset of spontaneous biofilm dispersal.

## 4. Conclusions

Altogether, the findings presented in this work suggest that NO elicits a metabolic burst in biofilm cells, and that a disruption in biofilm maturation occurs through the upregulation of endogenous central pathways that govern the transition from the biofilm to the planktonic lifestyle. Importantly, these data establish a relevant transcriptional framework that can serve as a benchmark for the future screening and mechanistic evaluation of candidate biofilm-dispersing agents.

## 5. Materials and Methods

### 5.1. Strains, Media and Culture Conditions

*Pseudomonas aeruginosa* PAO1 cultures were routinely grown overnight in LB (lysogeny broth) media at 37 °C, 200 rpm before incubation in fresh M9 media (9 mM NaCl, 22 mM KH_2_PO_4_, 48 mM Na_2_HPO_4_, 19 mM NH_4_Cl, 2 mM MgSO_4_, 100 µM CaCl_2_, 0.4% glucose, pH 7.0) at 37 °C with shaking.

### 5.2. Chemical Preparation and Storage

Spermine-NONOate (SP-NONO, CAT#0634655-16, (Z)-1-[N-[3 aminopropyl]-N-[4-(3-aminopropylammonio)butyl]-amino]diazen-1-ium-1,2-diolate) (Cayman Chemical, Ann Arbor, MI, USA) was dissolved in 10 mM NaOH to a final concentration of 10 mM and stored at −20 °C. Stock solutions were used within 3 months. 4-carboxy-TEMPO (C-TEMPO, CAT# 23139, 4-carboxy-2,2,6,6-tetramethyl-1-piperidinyloxy) (Cayman Chemical, Ann Arbor, MI, USA) was dissolved in water to a 10 mM solution. Only freshly made solutions were used in dispersal assays.

### 5.3. Biofilm Dispersal Assays

Biofilm formation and dispersal assays were performed as previously described [13]. Briefly, 10^7^ colony-forming units (CFU)/mL bacterial suspensions were prepared in M9 media and inoculated into a 24-well plate (Nunc, ThermoFisher Scientific, Waltham, MA, USA). Biofilms were grown at 37 °C, 180 rpm. At 4 h post-inoculation, SP-NONO (100 µM; Cayman Chemical), NaOH (100 µM) or 4-carboxy-TEMPO (500 µM; C-TEMPO; Cayman Chemical) were added to a final concentration of 100 µM or 500 µM, respectively, and incubated for another 15 min or 30 min. Wells were stained with 0.1% (*w*/*v*) crystal violet in 6.25% (*v*/*v*) methanol for 20 min, washed twice with 1 mL phosphate-buffered saline (PBS, Gibco, Franklin, TN, USA) and finally solubilised with ethanol (absolute). Optical density at 550 nm (OD_550_) was measured with a SPECTROStar Nano microplate reader (BMG LabTech, Ortenberg, Germany). Micrographs of stained biofilm were taken as described above.

### 5.4. RNA Sequencing and Analysis

Biofilms were incubated in tissue culture flasks as previously described with some adjustments [26]. Briefly, tissue culture flasks (Nunc, ThermoFisher Scientific, Waltham, MA, USA) were seeded with 10^7^ CFU/mL cells in 50 mL of M9 medium, and biofilms were grown at 37 °C with shaking (70 rpm). At 4 h, biofilms were treated with either SP-NONO (100 µM) or C-TEMPO (500 µM) for 15 min or 30 min. After treatment, the liquid phase was discarded, and the remaining attached cells were gently washed with PBS. Surface-attached cells were resuspended in a 1:2 mixture of PBS and RNA-protect solution (QIAGEN, Cat# 76506, Venlo, The Netherlands) using a cell scraper. Resuspended cells (~10^8^) were pelleted at 5000 g (10 min, 25 °C). RNA extraction was performed with the RNeasy mini kit (QIAGEN, Cat# 74104, Venlo, The Netherlands) following the manufacturer’s protocol. Samples were treated with DNase and subjected to RNA sequencing using DNBSEQ PE100 (BGI Genomics, Shenzhen, China). Cleaned reads were mapped to *P. aeruginosa* PAO1 chromosome (AE004091.2) using Bowtie2 v 1.50.2. Differentially transcribed genes between groups (untreated biofilms, biofilms treated with SP-NONO and biofilms treated with 4-carboxy-TEMPO; see Table S1) were identified with DESeq2 on Geneious Prime (v2024.07, Dotmatics, Boston, MA, USA). Principal component analysis (PCA) was conducted exclusively as an exploratory visualisation to assess overall transcriptomic similarity within biological replicates and to examine separation between treatment groups. For this purpose, the reads per kilobase of transcript per million mapped reads (RPKM) of genes with >|2-fold| transcription and *p*-value < 0.01 were employed. RPKM data was transformed into PCA scores using ClustVis [49], and subsequently plotted (Graphpad Prism 10.4.1, La Jolla, CA, USA).

## Figures and Tables

**Table 1:** Genes involved in energy generation that are differentially regulated by C-TEMPO and NO treatment in *P*. *aeruginosa* biofilms.

Gene	Fold Change—C-TEMPO ^a^	Fold Change—SP-NONO ^a^	Gene Product
Main anaerobiosis regulators			
*Anr*	−1.40	−1.60	transcriptional regulator Anr
*dnr*	**−2.16**	−1.64	transcriptional regulator Dnr
Oxidative phosphorylation			
*ccoP1*	1.33	0.98	cytochrome C oxidase cbb3-type subunit CcoP
*ccoQ1*	1.22	0.93	cytochrome C oxidase cbb3-type subunit CcoQ
*ccoO1*	1.16	0.94	cbb3-type cytochrome C oxidase subunit II
*ccoN1*	1.15	0.82	cbb3-type cytochrome C oxidase subunit I
*ccoP2*	**−9.30**	**−9.48**	cytochrome C oxidase cbb3-type subunit CcoP
*ccoQ2*	**−3.83**	**−4.51**	cytochrome C oxidase cbb3-type subunit CcoQ
*ccoO2*	**−8.92**	**−10.45**	cbb3-type cytochrome C oxidase subunit II
*ccoN2*	**−6.72**	**−8.18**	cbb3-type cytochrome C oxidase subunit Id
*coxA*	**2.08**	**2.12**	cytochrome C oxidase subunit II
*coxB*	**3.90**	**2.95**	cytochrome C oxidase subunit I
*cioA*	1.34	**2.15**	cyanide insensitive terminal oxidase
*cioB*	1.30	**2.01**	cyanide insensitive terminal oxidase
*cyoA*	1.09	1.80	cytochrome o ubiquinol oxidase subunit II
*cyoB*	1.06	1.27	cytochrome o ubiquinol oxidase subunit I
*cyoC*	1.26	1.01	cytochrome o ubiquinol oxidase subunit III
*cyoD*	1.31	0.87	cytochrome o ubiquinol oxidase subunit IV
*cyoE*	1.31	0.83	protoheme IX farnesyltransferase
Denitrification			
*nirN*	−1.25	**2.70**	cytochrome C
PA0510	−1.62	**3.55**	uroporphyrin-III C-methyltransferase
*nirJ*	−1.48	**3.67**	heme d1 biosynthesis protein NirJ
*nirH*	−1.10	**3.30**	heme d1 biosynthesis protein NirH
*nirG*	−1.80	**3.86**	heme d1 biosynthesis protein NirG
*nirL*	**−2.63**	**2.37**	heme d1 biosynthesis protein NirL
*nirD*	**−3.62**	1.76	heme d1 biosynthesis protein NirD
*nirF*	**−4.88**	1.54	heme d1 biosynthesis protein NirF
*nirC*	**−5.70**	**2.10**	cytochrome c55X
*nirM*	**−5.44**	**3.13**	cytochrome C-551
*nirS*	**−3.70**	**4.80**	nitrite reductase
*nirQ*	−0.76	**2.86**	denitrification regulatory protein NirQ
PA0521	−0.95	**6.67**	cytochrome C oxidase subunit
PA0522	−1.20	**6.08**	hypothetical protein
*norC*	−1.13	**45.90**	nitric oxide reductase subunit C
*norB*	−1.05	**31.34**	nitric oxide reductase subunit B
*norD*	**−2.88**	**7.54**	denitrification protein NorD
*nosR*	1.05	**8.26**	regulatory protein NosR
*nosZ*	1.02	**7.28**	nitrous-oxide reductase
*nosD*	0.95	**3.93**	copper-binding periplasmic protein
*nosF*	1.16	**4.09**	copper ABC transporter ATP-binding protein
*nosY*	0.88	**3.47**	membrane protein NosY
*nosL*	0.99	1.54	accessory protein NosL
Arginine deiminase pathway			
*arcD*	**−9.37**	**−8.02**	arginine/ornithine antiporter
*arcA*	**−12.14**	**−9.16**	arginine deiminase
*arcB*	**−11.07**	**−7.48**	ornithine carbamoyltransferase
*arcC*	**−3.08**	**−3.58**	carbamate kinase
Alcohol oxidation pathway			
*adhA*	**−6.79**	**−5.97**	alcohol dehydrogenase
*exaA*	−1.27	1.34	quinoprotein ethanol dehydrogenase
*exaB*	−1.39	1.84	cytochrome C550
*exaC*	**−2.23**	1.48	NAD^+^ dependent aldehyde dehydrogenase ExaC

^a^ relative to untreated biofilms. Bold indicates significant variation in gene expression.

**Table 2:** Genes uniquely upregulated during spontaneous and SP-NONO-induced dispersal of *P. aeruginosa* PAO1 biofilms.

Gene	Locus Tag	Fold Change—SP-NONO ^a^	Fold Change—Spontaneous Dispersal ^a^	Gene Product
Quorum sensing				
*agtA*	PA0604	4.63	3.20	ABC transporter
*agtB*	PA0605	3.61	2.27	ABC transporter permease
*agtC*	PA0606	2.36	2.10	ABC transporter permease
	PA1617	2.04	4.32	AMP-binding protein
*mexG*	PA4205	2.43	14.12	hypothetical protein
*mexH*	PA4206	2.48	15.14	resistance-nodulation-cell division (RND) efflux membrane fusion protein
*mexI*	PA4207	2.07	8.51	resistance-nodulation-cell division (RND) efflux transporter
Sulphur metabolism				
*cysW*	PA0281	−2.33	−3.01	sulfate transporter CysW
*cysT*	PA0282	−2.73	−2.81	sulfate transporter CysT
*cysP*	PA1493	−2.39	−2.04	sulfate ABC transporter substrate-binding protein
	PA3449	−3.23	−3.05	hypothetical protein
	PA3936	−2.16	−2.95	taurine ABC transporter permease
	PA3937	−2.45	−2.85	taurine ABC transporter ATP-binding protein
	PA3938	−2.57	−2.11	taurine-binding protein
*cysN*	PA4442	−2.50	−5.10	bifunctional sulfate adenylyltransferase subunit1/adenylylsulfate kinase
*cysD*	PA4443	−2.08	−3.43	sulfate adenylyltransferase subunit 2
ABC transporters				
*lhpK*	PA1255	2.60	4.38	trans-3-hydroxy-L-proline dehydratase
*lhpO*	PA1256	2.48	3.63	amino acid ABC transporter ATP-binding protein
*lhpM*	PA1258	2.41	2.25	ABC transporter permease
*nosF*	PA3394	4.08	3.89	copper ABC transporter ATP-binding protein
*opuC*	PA3891	3.56	7.06	ABC transporter ATP-binding protein
	PA4193	−2.25	−8.63	ABC transporter permease
	PA4194	−2.13	−4.14	ABC transporter permease
	PA4195	−2.08	−2.55	ABC transporter
	PA5095	2.10	2.55	ABC transporter permease
Two-component systems				
	PA0034	−2.00	−8.11	two-component response regulator
	PA0752	2.28	4.89	hypothetical protein
	PA0753	2.57	4.35	hypothetical protein
	PA0754	2.81	6.96	hypothetical protein
*dctA*	PA1183	44.63	7.57	C4-dicarboxylate transport protein
*pprB*	PA4296	2.14	8.57	two-component response regulator PprB
*tadB*	PA4301	2.00	23.10	type II secretion system protein TadB
*rcpA*	PA4304	2.11	28.25	type II/III secretion system protein
*rcpC*	PA4305	2.19	30.48	hypothetical protein
	PA4648	2.36	21.86	hypothetical protein
	PA4649	2.10	8.75	hypothetical protein
Alanine, aspartate and glutamate metabolism				
*davD*	PA0265	5.03	2.08	glutarate-semialdehyde dehydrogenase DavD
*davT*	PA0266	4.06	2.66	5-aminovalerate aminotransferase DavT
*ansB*	PA1337	2.33	3.36	glutaminase-asparaginase
	PA3356	3.66	2.57	hypothetical protein
	PA3758	3.39	2.13	N-acetylglucosamine-6-phosphate deacetylase
	PA3759	3.36	2.73	Aminotransferase
	PA3760	3.41	2.45	N-acetyl-D-glucosamine phosphotransferase system transporter
	PA5522	4.23	3.25	glutamine synthetase
	PA5523	5.50	2.91	Aminotransferase
Valine, leucine and isoleucine metabolism				
*braC*	PA1074	2.13	2.91	branched-chain amino acid ABC transporter substrate-binding protein BraC
*bkdB*	PA2249	2.10	3.56	branched-chain alpha-keto acid dehydrogenase complex lipoamide acyltransferase
*lpdV*	PA2250	2.69	2.93	branched-chain alpha-keto acid dehydrogenase complex dihydrolipoyl dehydrogenase
*leuD*	PA3120	−3.36	−3.18	isopropylmalate isomerase small subunit
*leuC*	PA3121	−2.53	−4.11	3-isopropylmalate dehydratase large subunit
	PA3417	2.66	13.09	pyruvate dehydrogenase E1 component subunit alpha
*ldh*	PA3418	2.19	17.88	leucine dehydrogenase
*ilvI*	PA4696	−2.07	−11.16	acetolactate synthase 3 catalytic subunit
Arginine and proline metabolism				
	PA4908	3.14	3.25	ornithine cyclodeaminase
	PA4909	2.68	3.27	ABC transporter ATP-binding protein
	PA4910	3.14	3.03	ABC transporter ATP-binding protein
	PA4911	3.97	3.07	branched-chain amino acid ABC transporter permease
	PA4912	2.77	2.30	branched-chain amino acid ABC transporter
	PA4913	2.01	2.14	ABC transporter
Bacterial chemotaxis				
	PA0173	3.12	4.17	chemotaxis response regulator protein-glutamate methylesterase
	PA0174	3.46	2.95	hypothetical protein
*cheR2*	PA0175	3.41	5.06	chemotaxis protein methyltransferase
*aer2*	PA0176	2.30	5.98	aerotaxis transducer Aer2
	PA0177	2.01	4.56	purine-binding chemotaxis protein
	PA0179	2.00	9.99	two-component response regulator
Porphyrin metabolism				
*cobP*	PA1278	−2.30	−5.70	bifunctional adenosylcobinamide kinase/adenosylcobinamide-phosphate guanylyltransferase
*cobU*	PA1279	−2.38	−5.03	nicotinate-nucleotide--dimethylbenzimidazole phosphoribosyltransferase
	PA1280	−2.33	−7.16	hypothetical protein
*cobV*	PA1281	−2.35	−6.06	adenosylcobinamide-GDP ribazoletransferase
	PA4088	2.27	2.64	Aminotransferase
	PA5523	5.50	2.91	Aminotransferase
Nicotinate and nicotinamide metabolism				
*pntAB*	PA0195.1	−2.23	−2.17	NAD(P) transhydrogenase subunit alpha
*pntB*	PA0196	−2.48	−2.35	pyridine nucleotide transhydrogenase subunit beta
*nadE*	PA4920	−2.51	−3.05	NAD synthetase
Pyrroloquinoline quinone biosynthesis				
*pqqA*	PA1985	2.51	14.03	coenzyme PQQ synthesis protein A
*pqqD*	PA1988	3.18	2.14	coenzyme PQQ synthesis protein D
*pqqE*	PA1989	3.73	2.85	coenzyme PQQ synthesis protein E
*pqqH*	PA1990	3.36	3.76	Peptidase

^a^ relative to untreated biofilms.

**Table 3:** Differential transcription of dispersal biomarkers induced by SP-NONO and C-TEMPO treatment in *P. aeruginosa* PAO1 biofilms.

Locus Tag	Fold Change—C-TEMPO ^a^	Fold Change—SP-NONO ^a^	Gene Product
PA0111	**5.15**	**6.62**	hypothetical protein
*cheR*2	1.44	**3.41**	chemotaxis protein methyltransferase
PA0743	1.32	1.83	NAD-dependent L-serine dehydrogenase
PA1353	1.70	1.47	hypothetical protein
*pqqA*	1.21	**2.52**	coenzyme PQQ synthesis protein A
*cdpR*	1.39	−1.01	transcriptional regulator
*amrZ*	1.16	1.40	alginate and motility regulator Z
*tadA*	**2.01**	**2.21**	ATPase TadA
*rcpA*	1.58	**2.12**	type II/III secretion system protein
*rcpC*	1.50	**2.18**	hypothetical protein
*flp*	**2.20**	**2.27**	type IVb pilin Flp
PA4523	−1.13	1.22	hypothetical protein
*cupE1*	1.79	**2.36**	fimbrial subunit CupE1
*cupE2*	1.46	**2.09**	fimbrial subunit CupE2

^a^ relative to untreated biofilms. Bold indicates significant variation in gene expression.

## Data Availability

The RNA-Seq data have been deposited in the NCBI Sequence Read Archive (SRA) database with accession code PRJNA1368986.

## Supplementary Materials

The following supporting information can be downloaded at: https://www.mdpi.com/article/10.3390/antibiotics15030278/s1, Figure S1: *P. aeruginosa* PAO1 growth kinetics under SP-NONO or C-TEMPO in M9; Table S1: Gene transcripts normalised by length and gene expression comparison between pairs; Table S2. Genes uniquely upregulated by SP-NONO and spontaneous dispersal but not C-TEMPO.

## Abbreviations

The following abbreviations are used in this manuscript:

NO	Nitric Oxide
SP-NONO	Spermine-NONOate
C-TEMPO	4-carboxy-TEMPO
RPKM	Reads per kilobase million

## References

[1] Flemming H.C., Wingender J. (2010). The biofilm matrix. Nat. Rev. Microbiol..

[2] Ciofu O., Tolker-Nielsen T., Bjarnsholt T., Jensen P.Ø., Moser C., Høiby N. (2011). Antibiotic tolerance and resistance in biofilms. Biofilm Infections.

[3] An S., Wu J., Zhang L.H. (2010). Modulation of *Pseudomonas aeruginosa* biofilm dispersal by a cyclic-di-gmp phosphodiesterase with a putative hypoxia-sensing domain. Appl. Environ. Microbiol..

[4] Sauer K., Cullen M.C., Rickard A.H., Zeef L.A.H., Davies D.G., Gilbert P. (2004). Characterization of nutrient-induced dispersion in *Pseudomonas aeruginosa* PAO1 biofilm. J. Bacteriol..

[5] Barraud N., Kjelleberg S., Rice S.A. (2015). Dispersal from Microbial Biofilms. Microbiol. Spectr..

[6] Chambers J.R., Cherny K.E., Sauer K. (2017). Susceptibility of *Pseudomonas aeruginosa* Dispersed Cells to Antimicrobial Agents Is Dependent on the Dispersion Cue and Class of the Antimicrobial Agent Used. Antimicrob. Agents Chemother..

[7] Barraud N., Storey M.V., Moore Z.P., Webb J.S., Rice S.A., Kjelleberg S. (2009). Nitric oxide-mediated dispersal in single- and multi-species biofilms of clinically and industrially relevant microorganisms. Microb. Biotechnol..

[8] Howlin R.P., Cathie K., Hall-Stoodley L., Cornelius V., Duignan C., Allan R.N., Fernandez B.O., Barraud N., Bruce K.D., Jefferies J. (2017). Low-Dose Nitric Oxide as Targeted Anti-biofilm Adjunctive Therapy to Treat Chronic *Pseudomonas aeruginosa* Infection in Cystic Fibrosis. Mol. Ther..

[9] Zhu X., Rice S.A., Barraud N. (2019). Nitric Oxide and Iron Signaling Cues Have Opposing Effects on Biofilm Development in *Pseudomonas aeruginosa*. Appl. Environ. Microbiol..

[10] Zhu X., Oh H.S., Ng Y.C.B., Tang P.Y.P., Barraud N., Rice S.A. (2018). Nitric Oxide-Mediated Induction of Dispersal in *Pseudomonas aeruginosa* Biofilms Is Inhibited by Flavohemoglobin Production and Is Enhanced by Imidazole. Antimicrob. Agents Chemother..

[11] Barnes R.J., Bandi R.R., Wong W.S., Barraud N., McDougald D., Fane A., Kjelleberg S., Rice S.A. (2013). Optimal dosing regimen of nitric oxide donor compounds for the reduction of *Pseudomonas aeruginosa* biofilm and isolates from wastewater membranes. Biofouling.

[12] Cai Y.M., Webb J.S. (2020). Optimization of nitric oxide donors for investigating biofilm dispersal response in *Pseudomonas aeruginosa* clinical isolates. Appl. Microbiol. Biotechnol..

[13] Forga X.B.I., Hong Y., Fairfull-Smith K.E., Qin J., Totsika M. (2025). Nitric oxide donor sodium nitroprusside serves as a source of iron supporting *Pseudomonas aeruginosa* growth and biofilm formation. Microbiol. Spectr..

[14] Barraud N., Schleheck D., Klebensberger J., Webb J.S., Hassett D.J., Rice S.A., Kjelleberg S. (2009). Nitric Oxide Signaling in *Pseudomonas aeruginosa* Biofilms Mediates Phosphodiesterase Activity, Decreased Cyclic Di-GMP Levels, and Enhanced Dispersal. J. Bacteriol..

[15] Matsuyama B.Y., Krasteva P.V., Baraquet C., Harwood C.S., Sondermann H., Navarro M.V.A.S. (2016). Mechanistic insights into c-di-GMP-dependent control of the biofilm regulator FleQ from *Pseudomonas aeruginosa*. Proc. Natl. Acad. Sci. USA.

[16] Liu S., Xu A., Xie B., Xin F., Dong W., Zhou J., Jiang M. (2022). Priority changes between biofilm exopolysaccharides synthesis and rhamnolipids production are mediated by a c-di-GMP-specific phosphodiesterase NbdA in *Pseudomonas aeruginosa*. iScience.

[17] Chua S.L., Liu Y., Li Y., Ting H.J., Kohli G.S., Cai Z., Suwanchaikasem P., Goh K.K.K., Ng S.P., Tolker-Nielsen T. (2017). Reduced intracellular c-di-GMP content increases expression of quorum sensing-regulated genes in *Pseudomonas aeruginosa*. Front. Cell. Infect. Microbiol..

[18] Arai H., Hayashi M., Kuroi A., Ishii M., Igarashi Y. (2005). Transcriptional regulation of the flavohemoglobin gene for aerobic nitric oxide detoxification by the second nitric oxide-responsive regulator of *Pseudomonas aeruginosa*. J. Bacteriol..

[19] Tucker N.P., D’Autréaux B., Spiro S., Dixon R. (2006). Mechanism of transcriptional regulation by the *Escherichia coli* nitric oxide sensor NorR. Biochem. Soc. Trans..

[20] Koskenkorva T., Aro-Kärkkäinen N., Bachmann D., Arai H., Frey A.D., Kallio P.T. (2008). Transcriptional activity of *Pseudomonas aeruginosa* fhp promoter is dependent on two regulators in addition to FhpR. Arch. Microbiol..

[21] Kuroki M., Igarashi Y., Ishii M., Arai H. (2014). Fine-tuned regulation of the dissimilatory nitrite reductase gene by oxygen and nitric oxide in *Pseudomonas aeruginosa*. Environ. Microbiol. Rep..

[22] Stamler J.S., Singel D.J., Loscalzo J. (1992). Biochemistry of Nitric Oxide and Its Redox-Activated Forms. Science.

[23] Volodarsky L.B., Reznikov V.A., Ovcharenko V.I. (1994). Synthetic Chemistry of Stable Nitroxides.

[24] De La Fuente-Núñez C., Reffuveille F., Fairfull-Smith K.E., Hancock R.E.W. (2013). Effect of nitroxides on swarming motility and biofilm formation, multicellular behaviors in *Pseudomonas aeruginosa*. Antimicrob. Agents Chemother..

[25] Reffuveille F., de la Fuente-Núñez C., Fairfull-Smith K.E., Hancock R.E.W. (2015). Potentiation of ciprofloxacin action against Gram-negative bacterial biofilms by a nitroxide. Pathog. Dis..

[26] Forga X.B.I., Fairfull-Smith K.E., Qin J., Totsika M. (2025). Transcriptional profiling of *Pseudomonas aeruginosa* biofilm life cycle stages reveals dispersal-specific biomarkers. bioRxiv.

[27] Kanehisa M., Goto S. (2000). KEGG: Kyoto encyclopedia of genes and genomes. Nucleic Acids Res..

[28] Belanger C.R., Dostert M., Blimkie T.M., Lee A.H.-Y., Dhillon B.K., Wu B.C., Akhoundsadegh N., Rahanjam N., Castillo-Arnemann J., Falsafi R. (2022). Surviving the host: Microbial metabolic genes required for growth of *Pseudomonas aeruginosa* in physiologically-relevant conditions. Front. Microbiol..

[29] Revelles O., Espinosa-Urgel M., Molin S., Ramos J.L. (2004). The davDT operon of *Pseudomonas putida*, involved in lysine catabolism, is induced in response to the pathway intermediate delta-aminovaleric acid. J. Bacteriol..

[30] Burns G., Brown T., Hatter K., Sokatch J.R. (1988). Comparison of the amino acid sequences of the transacylase components of branched chain oxoacid dehydrogenase of *Pseudomonas putida*, and the pyruvate and 2-oxoglutarate dehydrogenases of *Escherichia coli*. Eur. J. Biochem..

[31] Mattevi A., Obmolova G., Sokatch J.R., Betzel C., Hol W.G.J. (1992). The refined crystal structure of *Pseudomonas putida* lipoamide dehydrogenase complexed with NAD+ at 2.45 A resolution. Proteins.

[32] Gliese N., Khodaverdi V., Görisch H. (2010). The PQQ biosynthetic operons and their transcriptional regulation in *Pseudomonas aeruginosa*. Arch. Microbiol..

[33] Orillard E., Watts K.J. (2021). Deciphering the Che2 chemosensory pathway and the roles of individual Che2 proteins from *Pseudomonas aeruginosa*. Mol. Microbiol..

[34] Del Pozo J.L., Patel R. (2007). The challenge of treating biofilm-associated bacterial infections. Clin. Pharmacol. Ther..

[35] Lewis K. (2001). Riddle of biofilm resistance. Antimicrob. Agents Chemother..

[36] Reynolds D., Kollef M. (2021). The Epidemiology and Pathogenesis and Treatment of *Pseudomonas aeruginosa* Infections: An Update. Drugs.

[37] Vestby L.K., Grønseth T., Simm R., Nesse L.L. (2020). Bacterial biofilm and its role in the pathogenesis of disease. Antibiotics.

[38] Southey-Pillig C.J., Davies D.G., Sauer K. (2005). Characterization of temporal protein production in *Pseudomonas aeruginosa* biofilms. J. Bacteriol..

[39] Pant K., Palmer J., Flint S. (2025). Evaluation of single and multispecies biofilm formed in the static and continuous systems. Int. J. Food Microbiol..

[40] Rodesney C.A., Roman B., Dhamani N., Cooley B.J., Katira P., Touhami A., Gordon V.D. (2017). Mechanosensing of shear by *Pseudomonas aeruginosa* leads to increased levels of the cyclic-di-GMP signal initiating biofilm development. Proc. Natl. Acad. Sci. USA.

[41] Gamper M., Zimmermann A., Haas D. (1991). Anaerobic regulation of transcription initiation in the arcDABC operon of *Pseudomonas aeruginosa*. J. Bacteriol..

[42] Kawakami T., Kuroki M., Ishii M., Igarashi Y., Arai H. (2010). Differential expression of multiple terminal oxidases for aerobic respiration in *Pseudomonas aeruginosa*. Environ. Microbiol..

[43] Crocker A.W., Harty C.E., Hammond J.H., Willger S.D., Salazar P., Botelho N.J., Jacobs N.J., Hogan D.A. (2019). *Pseudomonas aeruginosa* Ethanol Oxidation by AdhA in Low-Oxygen Environments. J. Bacteriol..

[44] Feng Y., Adams E. (1977). Glutarate Semialdehyde Dehydrogenase of Pseudomonas. J. Biol. Chem..

[45] Chou H.T., Li J.Y., Lu C.D. (2013). Functional Characterization of the agtABCD and agtSR Operons for 4-Aminobutyrate and 5-Aminovalerate Uptake and Regulation in *Pseudomonas aeruginosa* PAO1. Curr. Microbiol..

[46] Valentini M., Storelli N., Lapouge K. (2011). Identification of C 4-dicarboxylate transport systems in *Pseudomonas aeruginosa* PAO1. J. Bacteriol..

[47] Underhill S.A.M., Cabeen M.T. (2022). Redundancy in Citrate and cis-Aconitate Transport in *Pseudomonas aeruginosa*. J. Bacteriol..

[48] Zhu X. (2018). Mechanisms of Nitric Oxide-Mediated Biofilm Dispersal in *Pseudomonas aeruginosa*. Ph.D. Thesis.

[49] Metsalu T., Vilo J. (2015). ClustVis: A web tool for visualizing clustering of multivariate data using Principal Component Analysis and heatmap. Nucleic Acids Res..

