# Supplementary material for: Nitric Oxide Donor Spermine-NONOate Elicits Endogenous Dispersal-Associated Transcriptional Responses to Promote Biofilm Dispersal in Pseudomonas aeruginosa
Source: Antibiotics (Basel). 2026 Mar 9;15(3):278. doi: 10.3390/antibiotics15030278 (PMC13024080; doi:10.3390/antibiotics15030278)
Supplement: Supplementary file 1 [file antibiotics-15-00278-s001.zip › Supplementary materials.pdf]

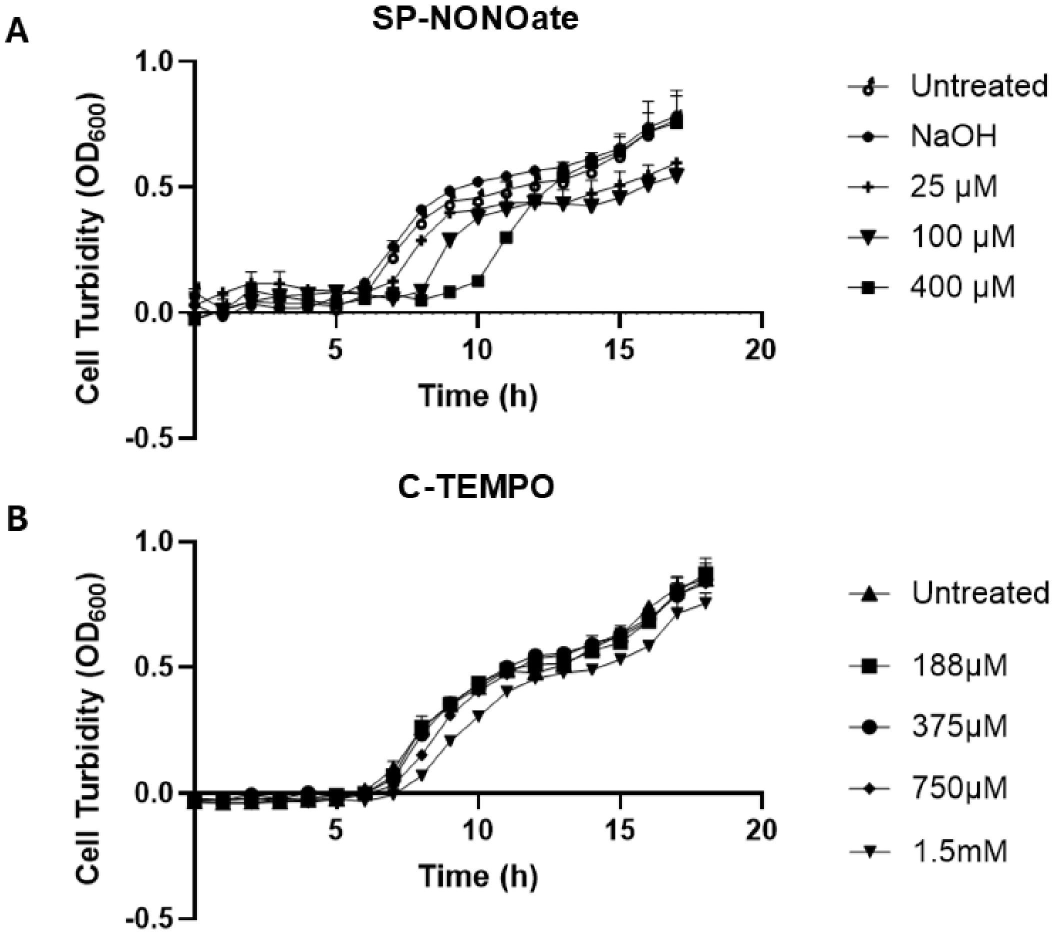

Figure S1. ***P. aeruginosa*** **PAO1 growth kinetics under SP-NONO or C-TEMPO in M9.** Overnight *P. aeruginosa* PAO1 cultures were diluted 1:1000 and subsequently seeded on 96-well plates in M9 supplemented with (A) SP-NONOate (25 - 400 µM) or NaOH (400 µM), and (B) C-TEMPO (188 µM – 1.5 mM). Cell turbidity was recorded by optical density at 600nm (OD_{600}) over 18 hours. 3 biological replicates were included. Means ± SD are represented in the graphs.

Table S2. Genes uniquely upregulated by SP-NONO and spontaneous dispersal but not C-TEMPO.

Locus tag	Name / predicted function	Log2 Fold-change SP-NONO vs Untreated	Log2 Fold-change Spontaneously dispersed cells vs Untreated
PA0034	two-component response regulator	-1.00	-3.02
PA0035	*trpA*	-1.78	-2.91
PA0036	*trpB*	-1.69	-3.55
PA0052	hypothetical protein	1.01	3.58
PA0109	hypothetical protein	1.12	3.25
PA0120	transcriptional regulator	1.10	1.53
PA0173	chemotaxis response regulator protein-glutamate methylesterase	1.64	2.06
PA0174	hypothetical protein	1.79	1.56
PA0175	chemotaxis protein methyltransferase	1.77	2.34
PA0176	*aer2*	1.20	2.58
PA0177	purine-binding chemotaxis protein	1.01	2.19
PA0179	two-component response regulator	1.00	3.32
PA0195.1	*pntAB*	-1.16	-1.12
PA0196	*pntB*	-1.31	-1.23
PA0265	*gabD*	2.33	1.06
PA0266	5-aminovalerate aminotransferase DavT	2.02	1.41
PA0281	*cysW*	-1.22	-1.59
PA0282	*cysT*	-1.45	-1.49
PA0299	*spuC*	2.50	1.41
PA0451	hypothetical protein	1.29	3.08
PA0462	hypothetical protein	1.06	1.19
PA0483	acetyltransferase	1.01	1.60
PA0520	*nirQ*	1.52	1.88
PA0543	hypothetical protein	1.20	2.78
PA0546	*metK*	-2.29	-1.12
PA0547	transcriptional regulator	-2.15	-1.20
PA0585	hypothetical protein	1.55	2.11
PA0586	hypothetical protein	1.31	2.88
PA0588	hypothetical protein	1.08	3.60
PA0604	ABC transporter	2.21	1.68
PA0605	ABC transporter permease	1.85	1.18
PA0606	ABC transporter permease	1.24	1.07
PA0752	hypothetical protein	1.19	2.29
PA0753	hypothetical protein	1.36	2.12
PA0754	hypothetical protein	1.49	2.80
PA0755	*opdH*	1.14	1.79
PA1074	*braC*	1.09	1.54
PA1183	*dctA*	5.48	2.92

PA1255	trans-3-hydroxy-L-proline dehydratase	1.38	2.13
PA1256	amino acid ABC transporter ATP binding protein	1.31	1.86
PA1258	ABC transporter permease	1.27	1.17
PA1278	*cobP*	-1.20	-2.51
PA1279	*cobU*	-1.25	-2.33
PA1280	hypothetical protein	-1.22	-2.84
PA1281	*cobV*	-1.23	-2.60
PA1337	*ansB*	1.22	1.75
PA1343	hypothetical protein	-1.24	-2.63
PA1493	*cysP*	-1.26	-1.03
PA1562	*acnA*	1.04	2.18
PA1592	hypothetical protein	1.24	3.01
PA1606	hypothetical protein	1.16	1.05
PA1617	AMP-binding protein	1.03	2.11
PA1742	amidotransferase	1.88	1.54
PA1743	hypothetical protein	1.08	1.16
PA1761	hypothetical protein	1.44	1.73
PA1985	*pqqA*	1.33	3.81
PA1988	*pqqD*	1.67	1.10
PA1989	*pqqE*	1.90	1.51
PA1990	*pqqH*	1.75	1.91
PA2140	metallothionein	1.02	3.17
PA2143	hypothetical protein	1.14	3.11
PA2151	alpha-1,4-glucan:maltose-1-phosphate maltosyltransferase	1.31	2.75
PA2249	*bkdB*	1.07	1.83
PA2250	*lpdV*	1.43	1.55
PA2274	hypothetical protein	1.08	3.15
PA2662	hypothetical protein	5.38	1.26
PA2664	*fhp*	6.71	1.90
PA2689	hypothetical protein	1.26	1.03
PA2691	hypothetical protein	4.49	2.32
PA2692	transcriptional regulator	1.41	1.47
PA2746a	hypothetical protein	1.12	2.38
PA2751	hypothetical protein	1.27	1.66
PA2776	hypothetical protein	3.05	1.58
PA2777	hypothetical protein	1.81	2.23
PA2778	hypothetical protein	1.01	1.81
PA2815	acyl-CoA dehydrogenase	1.08	2.89
PA2919	hypothetical protein	1.05	2.83
PA2929	hypothetical protein	-1.19	-1.32
PA2937	hypothetical protein	1.03	4.62
PA3041	hypothetical protein	1.26	1.96
PA3042	hypothetical protein	1.06	1.61
PA3049	*rmf*	1.03	5.18
PA3117	*asd*	-1.49	-2.66

PA3120	*leuD*	-1.75	-1.67
PA3121	*leuC*	-1.34	-2.04
PA3148	*wbpI*	-1.35	-1.96
PA3149	*wbpH*	-1.25	-2.33
PA3150	*wbpG*	-1.13	-1.75
PA3231	hypothetical protein	1.66	1.71
PA3356	hypothetical protein	1.87	1.36
PA3369	hypothetical protein	1.28	1.99
PA3371	hypothetical protein	1.42	1.94
PA3394	*nosF*	2.03	1.96
PA3417	pyruvate dehydrogenase E1 component subunit alpha	1.41	3.71
PA3460	acetyltransferase	1.25	2.70
PA3461	hypothetical protein	1.35	3.04
PA3510	hypothetical protein	1.32	2.04
PA3512	ABC transporter permease	1.06	1.37
PA3614	hypothetical protein	1.43	1.21
PA3723	FMN oxidoreductase	1.18	3.24
PA3727	hypothetical protein	-1.29	-1.22
PA3758	N-acetylglucosamine-6-phosphate deacetylase	1.76	1.09
PA3759	aminotransferase	1.75	1.45
PA3760	N-acetyl-D-glucosamine phosphotransferase systemtransporter	1.77	1.29
PA3891	ABC transporter ATP-binding protein	1.83	2.82
PA3922	hypothetical protein	1.06	2.34
PA3936	taurine ABC transporter permease	-1.11	-1.56
PA3937	taurine ABC transporter ATP-binding protein	-1.29	-1.51
PA3938	taurine-binding protein	-1.36	-1.08
PA4088	aminotransferase	1.18	1.40
PA4133	cbb3-type cytochrome C oxidase subunit I	-1.16	-2.16
PA4134	hypothetical protein	-1.03	-1.95
PA4153	2,3-butanediol dehydrogenase	1.23	1.12
PA4193	ABC transporter permease	-1.17	-3.11
PA4194	ABC transporter permease	-1.09	-2.05
PA4195	ABC transporter	-1.06	-1.35
PA4204	*ppgL*	1.43	2.07
PA4205	*mexG*	1.28	3.82
PA4206	*mexH*	1.31	3.92
PA4207	*mexI*	1.05	3.09
PA4288	transcriptional regulator	1.42	1.32
PA4296	*pprB*	1.10	3.10
PA4301	*tadB*	1.00	4.53
PA4304	*rcpA*	1.08	4.82
PA4305	*rcpC*	1.13	4.93
PA4311	hypothetical protein	1.39	3.23

PA4442	*cysN*	-1.32	-2.35
PA4443	*cysD*	-1.06	-1.78
PA4586	hypothetical protein	-1.00	-2.32
PA4648	hypothetical protein	1.24	4.45
PA4649	hypothetical protein	1.07	3.13
PA4680	hypothetical protein	1.15	1.57
PA4681	hypothetical protein	1.17	1.52
PA4696	*ilvI*	-1.05	-3.48
PA4813	*lipC*	1.42	1.04
PA4908	ornithine cyclodeaminase	1.65	1.70
PA4909	ABC transporter ATP-binding protein	1.42	1.71
PA4910	ABC transporter ATP-binding protein	1.65	1.60
PA4911	branched-chain amino acid ABC transporter permease	1.99	1.62
PA4912	branched-chain amino acid ABC transporter	1.47	1.20
PA4913	ABC transporter	1.01	1.10
PA4920	*nadE*	-1.33	-1.61
PA5015	*aceE*	-1.08	-1.21
PA5095	ABC transporter permease	1.07	1.35
PA5213	*gcvP1*	1.22	3.62
PA5274	*rnk*	-1.12	-2.91
PA5312	aldehyde dehydrogenase	2.10	1.22
PA5313	omega amino acid--pyruvate transaminase	2.31	1.58
PA5314	hypothetical protein	2.53	1.82
PA5521	short-chain dehydrogenase	1.14	1.38
PA5522	glutamine synthetase	2.08	1.70
PA5523	aminotransferase	2.46	1.54
